# Job satisfaction among medical assistants in German general practice: a qualitative study of social, structural and personal factors

**DOI:** 10.1186/s12875-026-03391-6

**Published:** 2026-05-22

**Authors:** Susanne Kersten, Elena Hohmann, Alexandra Piotrowski, Ann-Kathrin Lepper, Dorothea Dehnen, Michael Pentzek, Sabine Weissbach, Johanna Schweizer

**Affiliations:** 1 Chair of General Practice II and Patient-Centredness in Primary Care, Institute of General Practice and Primary Care (iamag), Witten/Herdecke University, Witten, Germany; 2 Institute of General Practice, Medical Faculty, University of Münster, Münster, Germany; 3 Institute of General Practice (ifam), Medical Faculty, University of Duisburg-Essen, Essen, Germany; 4 Institute of General Practice (ifam), Primary Care Research Group, Medical Faculty, University of Duisburg-Essen, Essen, Germany; 5 Medical Faculty, Institute of General Practice and Family Medicine (AM RUB), Ruhr University Bochum, Bochum, Germany; 6 North-Rhine Westphalian General Practice Research Network NRW-GPRN (Hausärztliches Forschungspraxennetz Nordrhein-Westfalen HAFO.NRW, https://hafo.nrw), Essen, Germany

**Keywords:** Medical assistants, Job satisfaction, Primary care, Qualitative research, Continuing education, Workforce, Germany

## Abstract

**Background:**

Medical assistants (MAs) are pivotal for access, coordination, and continuity in German primary care, yet workforce shortages and limited attention in research and policy persist. Prior studies quantify dissatisfaction and attrition drivers but offer little insight into how MAs who remain in practice experience job satisfaction. This study explores the social, personal and structural factors shaping MA job satisfaction in everyday work.

**Methods:**

We conducted 14 semi-structured telephone interviews with MAs from German general practices (November–December 2023), recruited via the research networks, the professional association (VMF e.V.) and teaching practice lists. Interviews lasted approximately 60 min and the interview guide covered practice organisation, communication, working conditions, and job satisfaction. Interview data were analysed using a rapid qualitative analysis approach. Key statements were summarised in a structured matrix aligned with interview topics and compared across cases. Categories were developed iteratively through team-based analysis and consensus meetings, resulting in three overarching domains influencing job satisfaction: social (team dynamics and leadership), personal (identity and motivation), and structural (roles, pay, digital infrastructure, and staffing). Ethics approval: University of Duisburg-Essen, Medical Faculty (23-11354-BO; 14 Nov 2023). Participants provided written consent and received a €75 expense allowance.

**Results:**

Three interrelated domains influenced job satisfaction.

(1) Social (team dynamics/leadership): Experiences ranged from respectful, supportive leadership to inattentive or inconsistent supervision; structured feedback and clear communication reduced errors and strain, while gossip/bullying and informal word-of-mouth channels increased frustration.

(2) Personal (identity/motivation): Satisfaction peaked when responsibilities matched training and skills were recognised and used. Emotional labour, especially in patient communication, was salient. Intrinsic motivation remained important, yet financial pressures increasingly shaped commitment.

(3) Structural (roles, pay, digital, staffing): Continuing education (CE) was valued, but many courses were only minimally reflected in pay scales and CE gains were not consistently implemented in contracts or roles. Pay was widely perceived as inadequate relative to responsibility, particularly during the pandemic. Digital infrastructure was often unreliable, especially in rural areas. Understaffing and irregular breaks and overtime contributed to overload. Substitution with non-clinical staff was viewed critically for clinically consequential tasks.

**Discussion:**

MA job satisfaction is co-produced by social, personal and structural conditions; the most salient burdens were emotional labour in patient contact and a misalignment between responsibility and compensation. Retention improves where leadership and communication routines are supportive, continuing education is translated into role/grade changes and visible use, and existing collective agreements are implemented consistently. Rather than new pay models, pragmatic fixes are needed, certificate-to-grade mapping, transparent role profiles, inflation-sensitive supplements, alongside dependable digital support. Delegation to non-clinical staff should exclude clinically consequential tasks (e.g. triage or telephone decisions).

**Conclusion:**

MA job satisfaction in German primary care emerges from interacting social, personal and structural conditions. Targeted actions across these domains are needed to sustain workforce capacity and patient-centred care.

**Supplementary Information:**

The online version contains supplementary material available at 10.1186/s12875-026-03391-6.

## What is already known

- MAs play a key role in ensuring access, coordination and continuity of primary care in Germany but have received limited attention in workforce research and policy.
- Previous surveys indicate that high workload, limited autonomy, lack of recognition and low pay contribute to dissatisfaction and workforce shortages.
- Existing qualitative studies mainly focus on psychosocial strain or reasons for leaving the profession, offering little insight into how job satisfaction develops among those who stay.

## What this paper adds

- This study shows that MAs’ job satisfaction arises from the interaction of social, personal and structural factors.
- It highlights that satisfaction is fostered by supportive leadership, clear communication and the meaningful use of skills, while emotional strain and misalignment between responsibility and compensation undermine it.
- It suggests that improving retention requires consistent implementation of existing pay frameworks, visible use of continuing education, and supportive team and communication structures.

## Introduction

Primary care systems around the world are under increasing pressure to maintain service quality amid growing personnel shortages [1].

This shortage is not coincidental but closely linked to the working conditions of non-physician health workers. Among these, medical assistants (MAs; in German *Medizinische Fachangestellte [MFA]*) are critical to ensure the continuity and effectiveness of care delivery. MAs perform organisational and medical functions and have close contact with patients, thus contributing significantly to access, coordination and continuity of care. In Germany, MAs represent one of the largest professional groups in outpatient health care [2].

Surveys and workforce reports highlight a decline in MA trainees and substantial challenges in filling open positions, particularly in primary care [3, 4]. Despite their central role, MAs remain underrepresented in workforce research, policy development and strategic planning [5].

Previous quantitative studies have shown that high workloads, limited autonomy and lack of recognition contribute to job dissatisfaction and professional exit among MAs [6], yet they offer limited insight into how these factors interact with professional identity and everyday experiences. Earlier qualitative research has primarily focused on psychosocial working conditions and preventive needs, highlighting high demands, low control, and limited rewards as central stressors for MAs [7].

More recently, Mambrey et al. [5] examined why MAs leave the profession, identifying heavy workload, insufficient career perspectives and lack of recognition as key drivers of attrition.

Survey data also suggest that while MAs are often satisfied with their workplaces and patient interactions, dissatisfaction is particularly pronounced regarding communication, leadership and opportunities for further training [8]. Another study concludes that in addition to structural conditions, factors such as leadership, task distribution and opportunities for further training are central predictors of job satisfaction among MAs [9].

Together, these findings indicate that job satisfaction arises from an interaction of structural and interpersonal conditions, but existing surveys provide limited insight into the subjective experiences and values that shape how these conditions are perceived.

Addressing this gap requires a qualitative perspective. Job satisfaction can be seen as a multidimensional construct, shaped not only by structural conditions but also by interpersonal relationships and personal meaning-making [6, 10]. Quantitative instruments often treat these dimensions in isolation and struggle to assess emotional, relational or value-based factors that strongly influence professional experience. A qualitative approach can therefore provide more nuanced insights into how MAs themselves make sense of satisfaction and dissatisfaction in their daily work.

In contrast to previous studies focusing on psychosocial strain [7] or exit decisions [5], our work investigates the mechanisms of job satisfaction among MAs who currently remain in practice, thereby complementing both strands of research.

The present study explores how MAs in German general practices experience and evaluate job satisfaction in their everyday work, with a particular focus on the interplay of structural, social and personal factors. By focusing on the perspectives of MAs themselves, this study seeks to illuminate how satisfaction or dissatisfaction arises from the interaction between social relationships in the workplace, structural conditions of employment, and personal values and motivations. The results are intended to inform practice-level strategies, policy decisions and workforce development initiatives aimed at improving job satisfaction and staff retention.

This perspective complements both strands of research by shifting the focus toward mechanisms that support professional sustainability.

This qualitative study was conducted within the North-Rhine Westphalian General Practice Research Network (NRW-GPRN) [11], a regional network of general practices in North Rhine-Westphalia. NRW-GPRN is one of six model networks established since 2020 as part of DESAM-ForNet [12], a nationwide initiative supported by the German Society of General Practice/Family Medicine (DEGAM; in German: *Deutsche Gesellschaft für Allgemeinmedizin und Familienmedizin*).

NRW-GPRN collaborates with MAs through an online MFA forum to identify and develop research topics relevant to everyday practice. A preceding survey on MA working conditions within this network highlighted several issues that could not be fully captured through quantitative data [13]. To explore these aspects in greater depth, we conducted a qualitative interview study focusing on MAs’ experiences of job satisfaction in everyday practice.

While previous research has largely examined dissatisfaction and attrition intentions among MAs, less is known about how those who remain in practice experience and sustain job satisfaction in their everyday work.

The central research question is: How do MAs in German general practices experience and evaluate the social, structural, and personal factors that influence their job satisfaction? By focusing on participants’ perspectives, the study aims to generate insights that go beyond standardised survey instruments.

The following section outlines the methodological approach, including participant recruitment, interview development and the steps of data analysis.

## Methods

### Study design and objectives

We designed this follow-up qualitative study to explore working conditions and job satisfaction among MAs in primary care using a rapid qualitative analysis approach. It complements a previous quantitative survey on MA working conditions, which is currently under review for publication. The latter survey had revealed several areas in need of deeper insight. The interview guide used in this qualitative study was newly developed specifically for this project in a participatory process with MAs. An English version of the main interview questions is provided in Supplementary File 1 (S-1_IG-main-questions_MA). Probing questions were used flexibly where relevant.

### Participant recruitment and sample

We conducted 14 semi-structured telephone interviews with MAs in Germany in November and December 2023. We chose the telephone format to ensure accessibility for all participants, regardless of geographic location, and to minimise participation barriers. This approach required only a phone line and a voice recorder.

We recruited five MAs through the HAFO.NRW research network and five through the mailing list and Instagram account of the professional association *“Verband medizinischer Fachberufe e.V.”* (VMF e.V.). Four additional participants registered by email; these were reached via the distribution list for teaching practices. Interview appointments were allocated on a first-come, first-served basis. Of the 21 MAs who registered for participation, 14 were included in the study. Participants were selected based on predefined criteria to ensure variation in.

 (1) involvement in research (yes/no),

 (2) affiliation with a professional association (yes/no),

 (3) practice setting (urban/rural), and.

 (4) varying lengths of professional experience.

Within these criteria, participants were enrolled on a first-come, first-served basis. The remaining MAs were placed on a waiting list in case additional interviews were required.

Participant characteristics are summarised in (Table 1).

VERAH (Versorgungsassistentin in der Hausarztpraxis) and EVA (Entlastende Versorgungsassistentin) are advanced qualification programmes for MAs enabling delegation of selected physician tasks, based on curricula of 200 + teaching units [14]. EVA is the regional designation used in North Rhine-Westphalia; the nationwide equivalent is NäPA (Nichtärztliche Praxisassistentin). According to the German Association of General Practitioners, over 15,400 VERAHs and approximately 12,000 EVAs/NäPAs are currently registered in Germany.

### Interview guide development and piloting

 We used a semi-structured guide to conduct the interviews, focusing on five key topics:

 (1) practice equipment,

 (2) practice organisation,

 (3) communication,

 (4) working conditions, and.

 (5) job satisfaction and work climate.

The topics were derived from the results of our previous survey and aimed to explore selected aspects in greater depth. Based on these survey findings, we drafted an initial interview guide.

The guide drew loosely on the language of the Consolidated Framework for Implementation Research (CFIR) [15, 16] to ensure coverage of organisational and individual factors (e.g., constructs related to inner setting and individual characteristics). However, CFIR was used only as a sensitizing heuristic during guide development and did not serve as a deductive coding framework in the analysis. To enhance comprehensibility and practical relevance, we piloted the guide using the Think-Aloud Method [17] with two experienced MAs from NRW-GPRN. This participatory step involved cognitive testing: MAs were asked to verbalise their understanding of each question, identify ambiguous wording, and suggest reformulations. The thematic areas were informed by findings from our preceding survey on MA working conditions, while the participatory piloting focused on improving question clarity, relevance, and comprehensibility. Based on their feedback, we adjusted language and removed items perceived as redundant or unclear. During piloting, MAs provided feedback on question clarity, relevance, and comprehensibility, which informed subsequent revisions.

### Interview procedure and data management

Two trained researchers conducted all interviews, each lasting approximately 60 min. Before the study, both interviewers received specific training in qualitative interviewing techniques, including how to address sensitive topics and maintain a neutral stance. To ensure consistency, the team conducted three internal training interviews to standardise approach and technique.

The interviewers had no prior personal relationship with the participants. Participants were informed about the interviewers’ professional background and the study’s purpose both in the recruitment email and again at the beginning of each interview. Interviews were conducted by telephone while participants were at their workplace or at home; to the best of our knowledge, no third persons were present. The Ethics Committee of the Medical Faculty of the University of Duisburg-Essen (23-11354-BO; 14.11.2023) approved the study.

All participants received written study information and gave written informed consent. Participation was voluntary, and participants were informed that they could withdraw at any time without providing a reason. The consent form also explained that no transcripts or audio recordings would be retained after the analysis and that all data would be deleted upon completion of the study.

All invited MAs chose to participate, and the interviews were completed in full. With participants’ consent, all interviews were audio-recorded. Immediately after each interview, the researchers documented key impressions and noteworthy points in field notes. All interviews were conducted in German. For publication, selected quotations were translated into English by the research team; translation prioritised preservation of both meaning and tone.

The analysis was conducted by a multidisciplinary team consisting of two doctoral researchers (one with a background in nursing, the other in psychology), one physician and one clinical study assistant who is a trained MA (*MFA*). All team members were affiliated with a university institute of general practice.

Participants received a €75 expense allowance. Funding was provided by the Federal Ministry of Education and Research (01GK1901H); compensation was not linked to responses.

### Data analysis

We applied a rapid qualitative analysis using summary templates and a cross-case matrix to synthesise interview data following Mathieson and Gale [18, 19]. This pragmatic approach supports timely yet rigorous findings in practice-based research [20], enables structured team-based analysis, and maintains analytic depth while reducing time to actionable results. Given the applied nature of our research question and the need to generate findings relevant for ongoing workforce discussions, a rapid approach was considered appropriate without compromising methodological rigour [19].

To structure the analysis, we developed a summary matrix aligned with the interview guide topics (not a theory-driven codebook). For each interview, we extracted key statements and summarised them in the matrix across all predefined topics. These summaries were based on field notes, transcript sections and repeated listening to the audio recordings to ensure accuracy and contextual understanding.

Two researchers independently analysed six interviews each and then reviewed each other’s summaries. In three consensus meetings, we jointly discussed the results, resolving discrepancies through collaborative dialogue rather than majority vote. Throughout this process, we critically reflected on our own assumptions to minimise bias.

As the analysis progressed, we rated and comparatively assessed the summarised content with regard to thematic relevance, salience and recurrence. We refined the matrix iteratively, added subcategories, clarified definitions and adjusted dimensions to better capture the complexity of the material.

### Detailed analytic procedure

Step 1 (Individual Summary): Each researcher listened to assigned interviews. Using a structured Excel template with columns corresponding to the guide themes, they extracted verbatim quotes and paraphrased summaries for each participant.

Step 2 (Cross-Check): Researchers exchanged their summaries and reviewed each other’s extractions, flagging discrepancies or alternative interpretations in tracked comments.

Step 3 (Consensus Meeting 1): The team convened to discuss flagged items. Discrepancies were resolved through collaborative dialogue: each researcher presented their interpretation, referenced the original audio, and the team negotiated a shared summary. No majority voting was used; rather, differences were explored until conceptual agreement was reached.

Step 4 (Pattern Identification): The consolidated summaries were re-read across cases. The team noted recurring patterns and began grouping content into preliminary categories.

Step 5 (Consensus Meeting 2): Preliminary categories were discussed and refined. Subcategories were added where variation was observed.

Step 6 (Domain Emergence): Through iterative comparison, three overarching domains – social, personal, and structural – emerged as organising principles that captured how participants framed their experiences.

Step 7 (Consensus Meeting 3): Final categories and domain structure were reviewed for coherence. The team verified that each domain was supported by multiple cases and that no significant content remained unassigned.

**Fig. 1:**
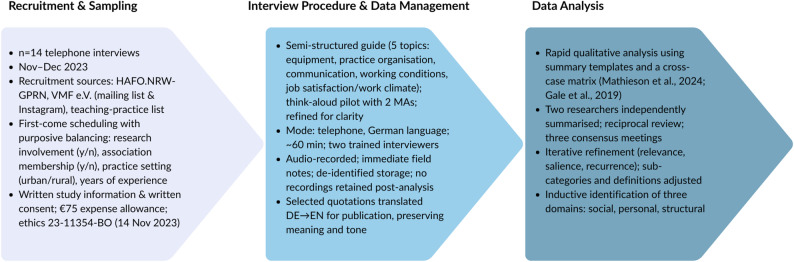
Data collection and analysis process

**Fig. 2:**
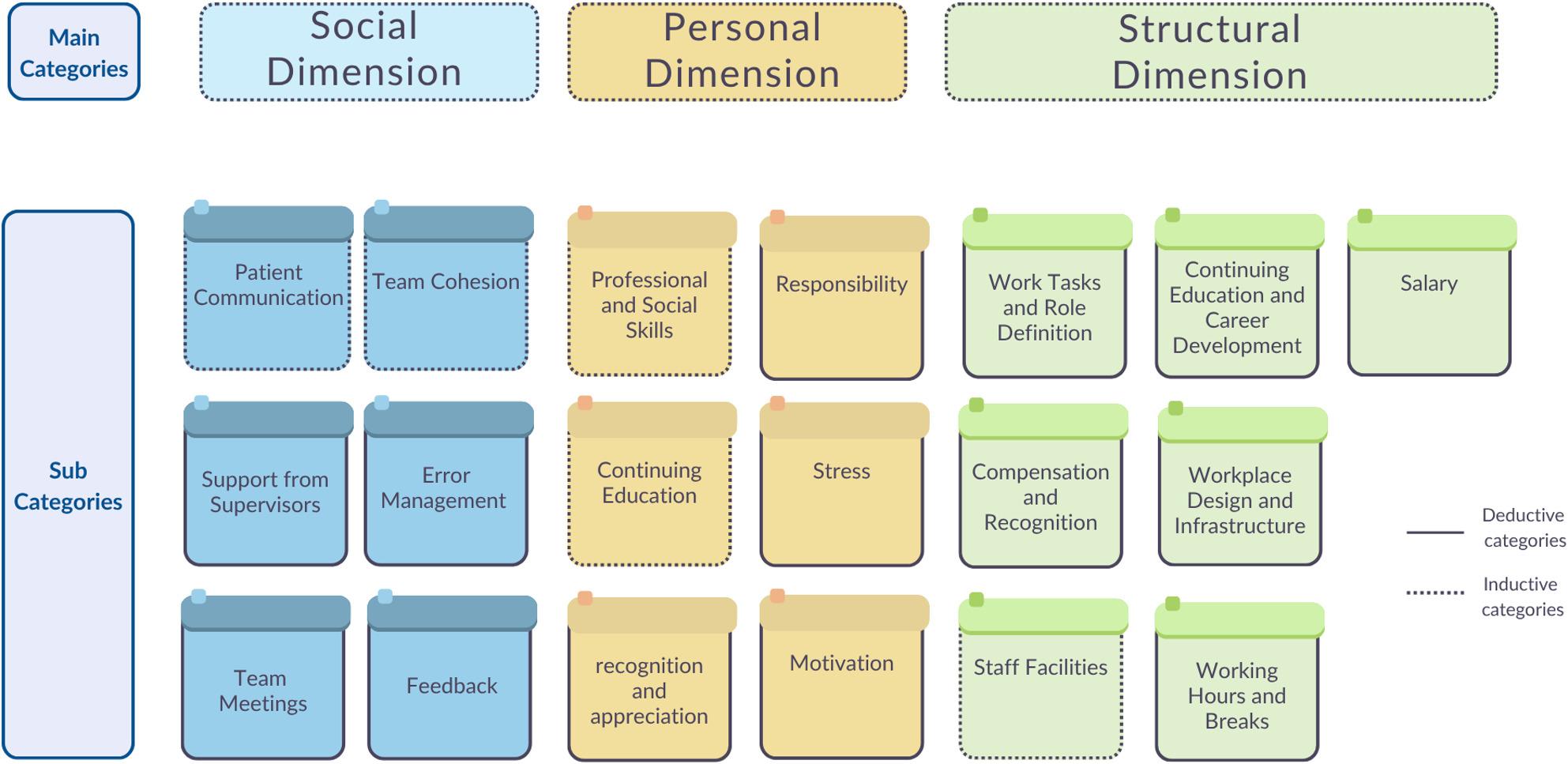
Categories

Our analytic approach combined inductive and deductive elements, consistent with hybrid thematic analysis [21, 22]. While the interview guide provided an initial deductive orientation based on prior survey findings, and some lower-level categories reflect this structure (Figs 1 and 2), the three overarching domains (social, personal, structural) emerged inductively from cross-case synthesis. Codes and sub-themes were generated directly from participant accounts rather than imposed from external theory. 

We developed a condensed coding framework that reflects the three thematic domains. This framework guided the reporting of results and is available as supplementary material (S-2_Coding-tree). 

### Quality assurance

To enhance the credibility and trustworthiness of the findings, several quality assurance strategies were employed:

1. Data triangulation: A purposive sampling strategy was applied to include MAs with varying backgrounds (e.g. research involvement, association membership, practice setting and experience), capturing a broad spectrum of perspectives.
2. Researcher triangulation: Two researchers independently conducted and analysed the data to minimise individual bias.
3. Reflexivity: After each analysis session, researchers wrote reflexive memos documenting subjective impressions, analytical uncertainties and potential biases. These memos were reviewed jointly to strengthen interpretative validity.
4. Transparent documentation: All analytic decisions and changes to the template were logged systematically for traceability.

## Results

### Social, personal and structural dimensions of job satisfaction

We interviewed *n* = 14 MAs from German general practices (median age 44.5 years; median contracted hours 28/week). Rural settings (*n* = 8) and solo practices (*n* = 7) were common. Overall, *n* = 13 reported any further training, most commonly vaccination (*n* = 4), practice/billing management (*n* = 3), VERAH/EVA (*n* = 3), endoscopy/anaesthesia (*n* = 2), diabetes care (*n* = 2), quality management (*n* = 2) and nutrition (*n* = 2) (Table 1).

**Table 1:** Sociodemographic and professional characteristics of participating medical assistants (*n* = 14)

Characteristic	Statistic	*n* = 14
Age	Median, range	44.5, 24–58
	Mean (SD)	42.8 (10.1)
	≤ 30 / 31–40 / 41–50 / 51–60	2 / 2 / 6 / 4
Contracted hours/week	Median, range	28, 20–38 (*n* = 13)
	Mean (SD)	29.7 (5.5) (*n* = 13)
	≤ 20 / 21–30 / 31–40	1 / 8 / 4 (*n* = 13)
Region	Rural / Urban	8 / 6
Practice type	Solo / Group / Shared / Medical centre (MVZ) / Not specified	7 / 2 / 2 / 1 / 2
Further training (any)	Yes / No	13 / 1
Selected training areas	Vaccination	4
	Practice/billing management	3
	VERAH/EVA	3
	Endoscopy/anaesthesia	2
	Diabetes care	2
	Quality management	2
	Nutrition	2

Cross-case synthesis identified three interrelated domains influencing job satisfaction: (1) social (interpersonal interactions and team dynamics) (2), personal (individual perceptions, needs, and motivation) and (3) structural (organisational and systemic work conditions).

Each of these domains comprises factors that either promote or hinder satisfaction in daily professional life. While distinct in content, the domains often intersect, illustrating the complexity of job satisfaction as an interdependent and context-sensitive phenomenon. This includes, for example, the interrelation of communication structures with leadership style, as well as the importance of continuing education and task distribution as both structural and personal drivers of satisfaction.

Participants described both burdens and sustaining aspects of their work. Supportive team relationships, meaningful patient contact, and recognition of competencies were frequently mentioned as factors that contributed to remaining in the profession despite everyday challenges.

#### Social dimension – interpersonal interactions in practice

Social and relational aspects were frequently described with notable emotional intensity by many participants. Interactions with patients, supervisors and team members shaped both positive and negative experiences and were often closely linked to participants’ sense of well-being at work.

Patient communication emerged as particularly challenging – especially during the COVID-19 pandemic. Many MAs recalled a significant rise in emotional strain due to verbal aggression, unrealistic patient expectations and a general decline in civility. These experiences contributed to psychological stress. One MA noted, *“It’s completely understandable that MAs are fleeing the profession*,* […]” (Interview 6)*, while another admitted she sometimes jokingly wished for a baseball bat at the reception desk to cope with the pressure.

Support from supervisors varied considerably. Some MAs described open and respectful relationships, while others reported inattentive or inconsistent leadership. One participant emphasised that the quality of supervisory relationships largely depended on the personalities involved—some worked well, others were more difficult. Across the board, participants identified good supervisory support as a crucial factor in their overall work environment.

Team cohesion and interpersonal dynamics also differed across practices. In some cases, participants highlighted a spirit of collaboration and mutual support. *“We work with each other instead of against each other*,* or whatever you want to call it; in other words*,* we actually work together as a team.” (Interview 3)*.

Others described more strained dynamics, including gossip or, in isolated cases, even workplace bullying. One participant mentioned that *“A bit of mobbing is part of it […]” (Interview 12)*, pointing to subtle but impactful conflicts.

Team meetings received mixed reviews. Some MAs valued them as essential tools for coordination and inclusion; others found them unstructured or redundant. Feedback ranged from *“absolutely essential” (Interview 6)* to *“not really necessary” (Interview 9).*

Regarding error management and feedback, some MAs valued regular performance reviews and reflective discussions, seeing them as crucial for improving processes and team climate.

*“I consider it a very important tool […] to assess satisfaction within the team on a small scale. To then initiate changes in the team meeting without naming names.” (Interview 1)*.

Others described informal communication paths and insufficient feedback systems, which sometimes led to repeated mistakes and unaddressed dissatisfaction. One MA noted that much of the communication occurred through word-of-mouth, increasing the risk of errors when not everyone was properly informed.

Appreciation was another recurring theme. One participant shared that during her last employee review, she expressed feeling underappreciated – adding that in some workplaces, the absence of complaints is still regarded as equivalent to praise.

These findings highlight the need for a structured and respectful communication culture that fosters team cohesion, ensures information flow and reduces interpersonal stress.

#### Personal dimension – perceptions, identity and motivation

The personal dimension of work refers to how individual perceptions, competencies, motivations and needs shape MAs’ experiences in general practice. Participants frequently mentioned their professional and social skills as important aspects of their role.

Professional and social skills were frequently mentioned as sources of pride. Many MAs expressed a strong sense of fulfilment when they could fully apply their skills across both clinical and administrative tasks and felt recognised for their abilities:

*“I’m satisfied when I’m allowed to do my work*,* the work I was trained for and to go beyond what’s expected.” (Interview 1)*.

At the same time, this sense of professional fulfilment was sometimes accompanied by a perception of being stretched beyond one’s limits. One MA described the increasing demands as overwhelming and admitted to working in a constant state of overload: *“We are working beyond our limits.” (Interview 8)*.

These contrasting experiences highlight the tension between professional engagement and emotional sustainability, particularly when appreciation and support are lacking.

Responsibility emerged as a multifaceted issue. Some participants welcomed additional duties and independent work as a sign of trust and professional respect, while others preferred to maintain defined roles, citing workload or lack of institutional support as reasons for hesitancy.

Continuing education was widely valued as a meaningful form of personal growth. However, participants frequently reported that such qualifications were undervalued or remained unused in practice, reducing their motivational effect.

*“I’ve done a lot of continuing education and would wish that it was actually used and appreciated in the practice.” (Interview 1)*.

Motivation profiles varied widely across participants. Intrinsic motivation, such as the desire to help others, remained an important driver. Several MAs, in turn, emphasised that financial compensation had become increasingly relevant to their ongoing commitment: *“For me*,* the only thing that counts anymore is what’s in my wallet.” (Interview 4)*.

These opposing statements illustrate growing tension between vocational ideals and economic reality.

Recognition and appreciation were recurring themes. Some MAs pointed out that they had not received even a basic COVID-19 bonus, while others felt that their work remained largely invisible to the management.

Several MAs also wished for more empathy and understanding from patients, identifying this as a key unmet need in their work environment. *“I wish patients were more patient and understanding […] that would really be a miracle.” (Interview 3)*.

Taken together, these findings reveal how personal motivation and satisfaction are deeply intertwined with recognition, meaningful use of skills, and a supportive work culture that addresses both emotional and material needs.

#### Structural dimension – organisational and systemic working conditions

Structural aspects of work refer to the objective framework conditions that shape the daily routines and responsibilities of MAs in general practice in Germany. These include task distribution, remuneration, opportunities for professional development, workplace infrastructure, scheduling and staffing levels. Across the interviews, participants described these conditions as both stabilising and burdening, depending on how they were implemented in practice.

Role clarity and task allocation varied considerably. Some MAs felt well-trained and confident in handling a wide range of responsibilities, including administrative and billing duties.

At the same time, several participants noted a noticeable shift in their roles: from patient-centred clinical tasks to more administrative or technical responsibilities. One MA reflected:

*“We used to do medicine with patients. Now it’s purely IT or business – just admin tasks.” (Interview 6).* Several interviewees associated this shift with changes in their professional role and a perceived reduction in the meaningfulness of their work.

 Continuing education was generally valued, especially when actively supported by supervisors. Several MAs emphasised that encouragement, protected time and opportunities to apply new skills in practice often had a stronger motivational effect than monetary incentives alone. *“Support from my boss for training makes me much more satisfied than a higher pay grade.” (Interview 1)*. While there are established training programs that lead to formal salary increases (e.g. as defined in collective wage agreements), participants described two distinct challenges: (1) many courses are only minimally or not at all mapped to the pay scale, and (2) even where formal upgrades exist, they are not consistently implemented or reflected in day-to-day roles. A lack of visible or immediate appreciation for newly acquired skills was described as demotivating: *“Many training courses are not covered by the collective agreement*,* and salary increases through training are minimal. That doesn’t create much incentive to do a lot of training and stuff like that. So why should you do it?” (Interview 12).* Positive examples also existed, with practices covering costs and offering regular opportunities: *“Training courses are always a good thing […] It’s great that they are offered regularly [in our practice]*,* all [costs] are covered by the boss.” (Interview 7).* Overall, this points less to a demand for rewards beyond the tariff and more to a call for consistent mapping, implementation and meaningful use of newly acquired competencies. Some also noted a lack of interest in further education among colleagues, which limited the broader impact at the team level.

Inadequate financial compensation was a recurring concern. Many MAs felt their salaries did not reflect the level of responsibility they carried – particularly during the pandemic, when stress and workload were described as exceptionally high. One MA reflected: *“…we were sitting there on the front line… I find that bitter*,* and that we were completely abandoned by the government… not seen*,* that’s more like it.” (Interview 5).* Others emphasised the need for structural reform in salary systems, including regular adjustments for inflation and performance. Without such changes, some questioned the long-term sustainability of their profession.

Participants described their workplace infrastructure in varying terms. Some participants described well-organised environments that supported efficient teamwork. Others reported technical challenges, particularly in relation to digitalisation. E-prescriptions and card readers were often described as unreliable, especially in rural areas, where the digital infrastructure was considered insufficient. One participant described this situation as being implemented without due consideration of local realities.

Regarding staff facilities, views varied. Some described break rooms as minimal or uncomfortable, while others pointed out the absence of basic amenities like a dedicated restroom for employees. One MA summed it up by saying that everything in the practice was well-equipped – except the break room: *“Everything is great*,* except for the area where the staff hangs out*,* which is a bit run down […] It would be nice to have more comfort for the medical assistants.” (Interview 10)*.

Working hours and break regulations also differed widely. Some participants praised fair systems for recording and compensating overtime, including options for time off. Others made a distinction between voluntarily staying longer and being forced to do so due to poor scheduling, which they found frustrating. Breaks were inconsistently practised: while a few described structured shift breaks as effective, others mentioned how unusual it still was to have officially recognised break times. One MA highlighted this by noting: *“After 25 years*,* it’s the first time I’ve had an official break. That’s never happened before.” (Interview 6)*.

Staffing levels were widely discussed. Many MAs identified understaffing as a key source of dissatisfaction and emotional exhaustion:

*“We can only be relieved if we have enough staff.” (Interview 1)*.

Attempts to integrate non-medical support staff were met with scepticism, particularly regarding tasks requiring medical judgment:

*“We tried non-medical staff. Didn’t work. You can’t make decisions on the phone without medical knowledge.” (Interview 6)*.

In sum, the interviews make it clear that organisational and systemic conditions strongly shape the work reality of MAs. Where supportive structures are in place, they contribute to satisfaction and motivation. Where they are lacking, they can lead to frustration, overload and, in some cases, the desire to leave the profession.

#### Reasons for staying

When asked directly why they continue working as MAs (Question 14), participants most frequently mentioned: (a) genuine enjoyment of interacting with patients and helping others, (b) collegial relationships and team cohesion, (c) flexible working hours that are compatible with family obligations, and (d) well-established routines and familiarity with their practice. Several participants also pointed to practical barriers to changing careers, such as the need for retraining or uncertainty regarding comparable employment opportunities. These responses largely mirrored the personal and social factors described above, suggesting that remaining in the profession is closely linked to the very conditions that promote job satisfaction.

## Discussion

The findings of this qualitative study underscore that job satisfaction among MAs in German general practices is shaped by a complex interplay of social, personal and structural factors. This extends earlier qualitative work that described psychosocial demands and low rewards as central risk factors for MAs [7], and complements recent research that explored the reasons why MAs leave the profession [5]. While Mambrey et al. (2024) [5] examined why MAs leave the profession, most previous qualitative studies have also sampled MAs currently working in practice. The distinctive contribution of our study lies therefore not primarily in sample composition, but in our analytic perspective: rather than retrospectively reconstructing exit decisions, we explored how satisfaction and dissatisfaction are negotiated in everyday work situations and which conditions enable MAs to remain committed to the profession despite recognised challenges. This prospective, retention-oriented lens complements attrition-focused research by identifying workplace mechanisms that stabilise professional commitment before concrete exit intentions emerge. Our findings suggest that the interplay of leadership quality, meaningful use of qualifications, and adequate recognition acts as a set of reinforcing conditions that can buffer strain and support continued engagement in the profession.

These factors represent potential leverage points for workplace-level interventions aimed at strengthening retention in primary care teams.

Furthermore, our results can be interpreted in the light of the Job Characteristics Model developed by Hackman and Oldham (1975) [23], which links job design dimensions such as task variety, autonomy and feedback to employee motivation and satisfaction. In this sense, our findings illustrate how classical job design dimensions are expressed in the specific context of general practice teams.

Our findings also confirm that, in addition to structural conditions, factors such as leadership, task distribution and opportunities for further training are central predictors of job satisfaction among MAs.

Our three interrelated domains emerged inductively from the interview material and reflect how MAs themselves describe the key factors influencing their work experience. This multidimensional perspective aligns with existing research and highlights the interplay of structural, interpersonal and individual factors in healthcare work satisfaction. Our findings add qualitative depth to prior survey-based studies that primarily quantified satisfaction levels among MAs.

While prior quantitative studies have documented associations between workload, recognition, and MA job satisfaction, our qualitative approach provides insight into how these factors interact in daily practice. By capturing perspectives of MAs during employment rather than retrospectively after exit, we reveal tensions between intrinsic motivation and structural constraints that may precede turnover.

The three-domain framework (social-personal-structural) offers an integrative lens that moves beyond single-factor explanations. For instance, we found that continuing education functions simultaneously as a structural condition (availability, cost coverage), a personal resource (skill development), and a social signal (recognition by supervisors). This interdependence has not been explicitly mapped in prior survey-based studies, which typically treat these dimensions as independent predictors.

While supportive team dynamics, opportunities for recognition and fair working conditions fostered engagement, the absence of these elements led to emotional strain, demotivation and, in some cases, the desire to leave the profession. Particularly striking were the emotional demands of patient communication, the desire for meaningful use of competencies, and the perceived mismatch between responsibility and compensation.

Although some themes, such as recognition, emotional stress or responsibility, recur across all three dimensions, we deliberately differentiate their roles: as relational experiences in the social domain, as subjective evaluations in the personal domain, and as externally shaped conditions in the structural domain. This analytic distinction reflects the complexity of job satisfaction as a context-sensitive and interdependent phenomenon. Our results are consistent with findings that emphasise the predictive role of leadership quality, task allocation and opportunities for professional development for MA job satisfaction [9].

We also observed divergent views in several areas, particularly regarding autonomy, team meetings and continuing education. While some MAs valued independence and flexible roles, others preferred clearly defined responsibilities. Similarly, experiences with team communication and leadership ranged from highly positive to deeply frustrating. These differences highlight the heterogeneity within the professional group and suggest that job satisfaction is not only context-dependent, but also influenced by individual preferences, practice culture and team dynamics.

In line with prior studies highlighting the importance of interpersonal climate in healthcare settings, our results underscore the role of social relationships as both resources and stressors [24]. Patient communication was described not only as technically challenging but also as emotionally demanding — particularly in pandemic contexts marked by aggression and incivility. These findings support the concept of “emotional labor” [25] as a core yet largely invisible component of MA work. However, in contrast to emotional labour research in nursing, we observed a distinct lack of institutional support structures in MA settings, indicating a potential need for improved support structures in team communication, stress management and supervision, particularly in light of the emotional demands described by participants.

The personal dimension highlights how MAs experience their roles at the intersection of autonomy, recognition and professional identity. Satisfaction was highest when responsibilities matched training and when efforts such as continuing education were acknowledged and used. These findings are consistent with studies which emphasise better salary, appreciation and more involvement in health policy decisions [7, 26]. Furthermore, participants expressed growing frustration over economic pressures. The frequent mention of financial strain suggests a shift in professional values under deteriorating working conditions, suggesting that extrinsic motivators such as salary are increasingly displacing intrinsic ones like patient care motivation – a trend with potential consequences for long-term retention strategies.

Structurally, job satisfaction was shaped by task distribution, compensation, digital infrastructure and staffing levels. Several participants described a shift from clinical to administrative roles, leading to a perceived loss of professional identity. This resonates with recent findings that highlight the opportunities and challenges of delegating medical tasks to MAs as part of securing primary care, while also raising questions about workload distribution and practice organisation [27]. It also aligns with literature on role erosion among support staff in healthcare [28] and signals a need for clearer task delineation and participatory role development. Inadequate pay, especially during periods of heightened responsibility such as the COVID-19 pandemic, was consistently framed as a form of devaluation. These findings mirror broader trends in healthcare where compensation fails to reflect the scope and complexity of work, contributing to attrition risks [5, 29, 30]. 

### Implications for practice

The findings point to several actionable implications for healthcare management, professional development and policy.

First, the emotional labour inherent in MA roles, particularly in high-stress contexts such as the winter season with many infections, requires explicit organisational recognition. Based on participants’ descriptions of emotional strain, practices should consider implementing support structures such as structured breaks, access to team reflection or supervision, and de-escalation training – strategies that have shown to support staff well-being in similar healthcare contexts [24]. Emotional resilience should not remain the sole responsibility of individuals.

Second, based on our findings, continuing education should not remain a mere formality but be meaningfully integrated into practice roles and career development. While some training pathways already include transparent links to salary progression, participants emphasised that the practical application and recognition of newly acquired skills often remain limited. Supervisors can play a key role by actively supporting further training and by ensuring that new qualifications are reflected not only in remuneration, where applicable, but also in task allocation and professional appreciation. Strengthening the connection between continuing education, role development and visible recognition may help improve both motivation and retention.

Third, leadership in general practices should include basic training in team communication, conflict resolution and employee recognition. The data show that supervisory behaviour strongly affects team climate, motivation and perceived appreciation. Leadership development programs—particularly for practice owners and senior staff—can help foster a culture of transparency and mutual respect.

Fourth, many participants described their salaries as inadequate, especially given the stress caused by the pandemic and the increasing complexity of their tasks. Rather than proposing entirely new payment structures, our data points to implementation gaps within existing collective bargaining agreements. Training is sometimes only minimally or not at all reflected in the salary scale, and when further training has been completed, it is not consistently implemented in contracts or taken into account in the distribution of tasks. It would be desirable to convert certificates into collective agreement-related upgrades, have transparent role profiles that align additional responsibility with the corresponding job group, and provide inflation-sensitive allowances for periods of increased responsibility. This approach can help to remedy the perceived imbalance between responsibility and compensation and promote long-term employee retention. It requires consistent assignment, implementation and visible use of newly acquired skills.

Fifth, digital tools and workflows need to be implemented with attention to local conditions and end-user realities. Especially in rural areas, many MAs described the digital infrastructure as unreliable or poorly adapted to their work environment. While feedback from the pilot regions has been integrated into system adjustments (e.g. in regard to user-oriented designs and participatory implementation strategies), practices will benefit above all from reliable support (e.g. a direct hotline) in the event of system failures and low-threshold regular training courses.

Finally, the integration of non-clinical staff should be considered with caution. While task delegation can help buffer staffing shortages, participants viewed the transfer of clinically consequential activities (e.g. telephone-based decision-making) critically. Where the use of non-clinical support is unavoidable, roles should be co-designed with MAs, communicated transparently and restricted to tasks that do not require clinical judgment. Given the relevance for workforce strategies in health care, we foreground this as a priority for communication and role clarity by practice owners.

### Strengths and limitations

This study offers a rich qualitative insight into the lived experiences of MAs in German general practice settings. By organising the findings across three dimensions (personal, social and structural) it captures the complexity of job satisfaction as a multidimensional construct. These findings are supported by a qualitative thematic review which highlights that emotional labour in healthcare involves intrapersonal, collegial and organisational dimensions, and underscores the need for proactive support and training structures.

Methodological strengths include purposive sampling to ensure variation across practice contexts and participant characteristics, a multidisciplinary analysis team (including a trained MA), researcher triangulation with consensus meetings, reflexive memos, and piloting of the interview guide.

Adherence to reporting standards (e.g. COREQ) and the provision of anonymised analytic instruments (coding tree, key quotes) further enhance transparency and credibility.

However, the study also has limitations. The sample, while diverse in age and employment setting, may not fully represent the broader MA workforce across all regions or practice types; recruitment channels may have over-represented more engaged participants. Social desirability bias cannot be excluded, particularly regarding statements about team cohesion or personal motivation. The telephone mode, while improving access and feasibility, constrained the capture of emotional nuances and, by design, non-verbal communication could not be observed, which may have limited contextual interpretation. We sought to mitigate this through repeated listening and systematic field notes. Because interviews were conducted in German and quotes were translated into English for reporting, minor shifts in meaning are possible. A rapid qualitative approach using structured Excel templates was chosen for practice relevance and timeliness; while pragmatic, this may limit analytic depth compared with full code-by-code MAXQDA workflows. The expense allowance (€75) could have influenced self-selection, though compensation was not contingent on responses. We did not include observational data or triangulate with other stakeholder groups (e.g. practice owners, GPs) within this study, which may limit perspective breadth.

The interview guide included a specific question about reasons for remaining in the profession (question 14), but responses often overlapped with other themes and were thus integrated into the broader domains rather than reported separately.

Additionally, the study was conducted within the specific context of the German healthcare system. Given international differences in role profiles, training pathways and primary care structures, the transferability of findings to other countries is inherently limited. As a qualitative study, findings are not intended to be statistically generalisable; rather, they offer depth of understanding and hypothesis generation for future research.

### Future research

Further research could explore how the interplay between social, personal and structural factors affects job satisfaction across different roles in primary care. While our data suggest that satisfaction emerges from the dynamic balance between these domains, future studies could investigate how the disruption of one domain — such as weak leadership or lack of recognition — affects the overall perception of work.

Quantitative approaches may help identify how widespread such dynamics are, particularly regarding emotional labour, task identity and the practical impact of continuing education.

Longitudinal designs could also track how workplace conditions evolve over time and whether targeted interventions (e.g. leadership training, feedback systems) lead to sustainable improvements.

## Conclusion

This study highlights how job satisfaction among MAs in German general practice is shaped by the interplay of social, personal and structural factors. On a social level, respectful communication, team cohesion and supportive leadership emerged as critical drivers of job satisfaction, while interpersonal tensions and emotional strain, especially in patient contact, posed major challenges. At the personal level, MAs derived motivation from applying their skills meaningfully and being recognised for their contributions, yet experienced frustration when autonomy and continuing education were not matched by institutional support. On a structural level, task distribution, salary, digital infrastructure, and staffing conditions directly influenced satisfaction, identity and retention. Improving MA job satisfaction thus requires targeted action in all three dimensions – relational, individual and systemic – to strengthen the resilience and sustainability of primary care teams.

Addressing these challenges is essential not only for improving work satisfaction, but also for safeguarding the future of patient-centred primary care.

While this study focuses on the German context, the findings may also offer impulses for exploring job satisfaction among comparable roles in other primary care systems.

## Supplementary Information

Supplementary Material 1.

Supplementary Material 2.

Supplementary Material 3.

## Data Availability

The qualitative datasets (audio recordings and verbatim transcripts) generated and analysed during this study are not publicly available due to privacy and ethical restrictions. In line with ethics approval from the Ethics Committee of the Medical Faculty, University of Duisburg-Essen (ref. 23-11354-BO, 14 Nov 2023) and participants’ informed consent, audio files and transcripts were not retained after completion of the analysis and have been deleted. Consequently, no primary individual-level data can be shared. The article includes anonymised illustrative quotations. The condensed coding framework and the cross-case summary matrix template used in the rapid qualitative analysis are provided as Supplementary material. Methodological queries may be directed to the corresponding author.

## Abbreviations

GP practices: General practice (GP) practices
MAs: Medical assistants, qualified non-physician clinical staff working in German general practice
NäPa: Non-physician practice assistant (Nicht-ärztliche Praxisassistent/in)
VERAH: Care assistant in general practice (Versorgungsassistent/in in der Hausarztpraxis)
NRW-GPRN: North-Rhine Westphalia General Practice Research Network
VMF e.V.: Association of Medical Professionals
