# Supplementary material for: Job satisfaction among medical assistants in German general practice: a qualitative study of social, structural and personal factors
Source: BMC Prim Care. 2026 May 22;27:214. doi: 10.1186/s12875-026-03391-6 (PMC13220601; doi:10.1186/s12875-026-03391-6)
Supplement: Supplementary file 2 — Supplementary Material 2. [file 12875_2026_3391_MOESM2_ESM.docx]

| Thematic Domain | Subdomain | English translation | Used in text? | Interview |
| --- | --- | --- | --- | --- |
| Social dimension | Patient communication | “It's completely understandable that MAs are fleeing the profession.” | Yes | Interview 6 |
|  |  | “During the pandemic, there were times [...] when I thought I needed a baseball bat at the registration desk [...] it was unbelievable.” | Yes paraphrased | Interview 6 |
|  | Support from supervisors | “Our bosses… you get along with one, but sometimes not with the other, it’s on and off actually, but overall it’s quite good.” | No | Interviews 7 |
|  |  |  |  |  |
|  | Team cohesion and interpersonal dynamics | “We work with each other instead of against each other, or whatever you want to call it; in other words, we actually work together as a team.” | Yes | Interview 3 |
|  |  | “A bit of mobbing is part of it [...].” | Yes | Interview 12 |
|  |  | “Gossip happens within a normal range, but it never gets really harsh or personal.” | No | Interview 3 |
|  | Team meetings | “For me, team meetings are absolutely essential – without team meetings, no practice can function.” | Yes | Interview 6 |
|  |  | “There’s no real need for them to take place […] it’s just repeating what was discussed last time.” | Yes | Interview 9 |
|  | Error management and feedback | “I consider it a very important tool […] to assess satisfaction within the team on a small scale. To then initiate changes in the team meeting without naming names.” | Yes | Interview 1 |
|  |  | “A lot of things are passed on only by word of mouth. Often the same mistake is then made by a second person who hasn’t been sufficiently informed about it.” | Yes paraphrased | Interview 5 |
|  |  | “Error management always means self-criticism and growth.” | No | Interview 6 |
|  | Appreciation | “I also expressed that in the last employee review, where I said that appreciation is not expressed often enough. Like in other workplaces too: not being criticized is praise enough.” | Yes paraphrased | Interview 5 |
|  |  |  |  |  |
| Personal dimension | Professional and social skills | “I’m satisfied when I’m allowed to do my work, the work I was trained for and to go beyond what’s expected.” | Yes | Interview 1 |
|  |  | “We are working beyond our limits.” | Yes | Interview 8 |
|  | Responsibility | “Well, I think it’s nice, it’s independent work. Also trust […]” | No | Interview 9 |
|  |  | “Do I want more responsibility? 'Actually, no'.” | No | Interview 7 |
|  | Continuing education | “I’ve done a lot of continuing education and would wish that it was actually used and appreciated in the practice.” | Yes | Interview 1 |
|  |  |  |  |  |
|  | Motivation profiles | “For me, the only thing that counts anymore is what’s in my wallet.” | Yes | Interview 4 |
|  |  | “Work with the people, for the people.” | No | Interview 1 |
|  |  | “I don’t like monotonous work.” […]  “I just like it […] the hustle and bustle around me.” […]  “You never stop learning.” | No | Interview 7 |
|  | Recognition and appreciation | “COVID bonus […] we didn’t get a cent.” | Yes paraphrased | Interview 10 |
|  |  | “Because many people don’t even know what MAs actually do at the front desk. It’s not just answering phones and smiling.” | No | Interview 6 |
|  | Stress factors | “So exhausted after work that sometimes I need a couple of hours of sleep first.” | No | Interview 9 |
|  |  | “The patients place a lot of demands on us/on the MAs. ‘Pre-doctor’ – it’s already gone kind of crazy.” | No | Interview 2 |
|  | Miracle question / wish | “I wish patients were more patient and understanding […] that would really be a miracle.” | Yes | Interview 3 |
| Structural Dimension | Role clarity and task allocation | “We used to do medicine with patients. Now it’s purely IT or business – just admin tasks.” | Yes | Interview 6 |
|  |  | “[Many tasks, e.g. billing] we can do. We are very well trained. Just let us do our job. That’s what I would wish for in the future.” | Yes paraphrased | Interview 1 |
|  | Continuing education | “Support from my boss for training makes me much more satisfied than a higher pay grade.” | Yes | Interview 1 |
|  |  | “Many training courses are not covered by the collective agreement, and salary increases through training are minimal. That doesn't create much incentive to do a lot of training and stuff like that. So why should you do it?“ | Yes | Interview 12 |
|  |  | “Training courses are always a good thing […] It's great that they are offered regularly [in our practice], all [costs] are covered by the boss.” | Yes | Interview 7 |
|  |  | “Unfortunately, my colleagues just aren’t interested. […] No, none of them want any of that.” | No | Interview 8 |
|  | Inadequate financial compensation | “...we were sitting there on the front line... I find that bitter, and that we were completely abandoned by the government... not seen, that's more like it.” | Yes | Interview 5 |
|  |  | “Politics finally has to act so that salaries can be paid according to performance – with annual adjustment of salaries – otherwise it won’t work.” […]  “[…] [that’s one] reason why many say: for that money, to expose myself to that level of stress, with that workload, and to be insulted on top of that – who would do that?” | No | Interview 6 |
|  | Workplace infrastructure | “Very structured, clear workstations; a back office where two people can make calls […] a prescription phone; reception area where two employees sit – one answering the phone, the other accompanying patients into the rooms or with the doctor.” | No | Interview 6 |
|  |  | “Still a problem in the practice: the e-prescription doesn’t work at all, the e-sick note only sluggishly. It starts with the card readers that crash all the time and make life difficult for us. But that’s something the practice itself can’t really work on, that’s just TI [= telematics infrastructure] again […] pushing a project forward without even looking at how it works in rural areas. […] And when e-prescriptions become mandatory in January […] that’s also an issue that really stresses out a lot of MAs.” | Yes paraphrased | Interview 6 |
|  |  |  |  |  |
|  | Staff facilities | “Everything is great, except for the area where the staff hangs out, which is a bit run down [...] It would be nice to have more comfort for the medical assistants.” | Yes | Interview 10 |
|  | Working hours and break regulations | “After 25 years, it’s the first time I’ve had an official break. That’s never happened before.” | Yes | Interview 6 |
|  |  | “Regarding breaks we’re really well structured, because we don’t have long lunch breaks. So we’re there continuously from 6:45 am to 5:00 pm, working in shifts. The colleagues who are on duty all have their breaks accordingly. That’s really well structured, it fits, there’s really nothing much to complain about […] I wouldn’t know how you could improve that.” | No | Interview 1 |
|  | Staffing levels | “We can only be relieved if we have enough staff.” | Yes | Interview 1 |
|  |  | “We tried non-medical staff. Didn’t work. You can’t make decisions on the phone without medical knowledge.” | Yes | Interview 6 |

***Tab. 3: Coding tree with MA key quotes***
