# Supplementary material for: Job satisfaction among medical assistants in German general practice: a qualitative study of social, structural and personal factors
Source: BMC Prim Care. 2026 May 22;27:214. doi: 10.1186/s12875-026-03391-6 (PMC13220601; doi:10.1186/s12875-026-03391-6)
Supplement: Supplementary file 3 — Supplementary Material 3. [file 12875_2026_3391_MOESM3_ESM.docx]

Interview Guide – Main Questions

***Job satisfaction among medical assistants in German general practice***

Version 2.0 | November 13, 2023

1. What is it like for you in practice? When you think about your work, what do you associate with satisfaction and what with dissatisfaction?
2. How do you like the design of your workplace? Describe your GP practice. I am referring specifically to the facilities and the premises.
3. What changes or improvements would you recommend in the design of the workplace or break rooms?
4. Now I am interested in how you organize your working hours. How is this regulated in your practice?
5. How do you experience the interaction within your MFA team in your practice?
6. How is the relationship with your boss?
7. How does your team deal with mistakes?
8. Is there a structured error management system in the team?
9. Are there also regular feedback meetings/performance reviews *(praise/recognition/personal development)*?
10. Financial incentives (coronavirus bonuses, salary increases) and decision-making authority, Increasing decision-making authority: What is the situation regarding responsibilities in your practice? Are there any medical tasks that are delegated to medical assistants, e.g., in the areas of taking patient histories, initiating and performing diagnostic tests, issuing (including by telephone) certificates of incapacity for work?
    What do you think about this? What is your opinion?
11. Does your job satisfaction or dissatisfaction have anything to do with financial aspects?
12. How much “more” would you like to earn? Cave: brutto/netto!
13. In your opinion, what additional financial incentives or bonuses would increase the motivation and satisfaction of medical assistants?
14. Reflecting on the topics mentioned, what keeps you in your job?
15. What are the key factors for you to enjoy working as an MFA?
16. Imagine: if you woke up tomorrow and all your problems were solved, what would be the first thing you would notice?
17. What year were you born?
18. How long have you been working as an MFA?
19. How many hours per week do you work?
20. Do you have any additional qualifications (NäPa, VERAH, etc.)?
21. How long have you been working at your current practice?
22. What is your practice like (private practice, group practice, location, etc.)?
23. Does the practice belong to the General Practice Research Network North-Rhine Westphalian?
24. Gender

**List of Abbreviations**

GP practices = **General practice (GP) practices**

MAs = qualified non-physician clinical staff working in German general practice

NäPa = Non-physician practice assistant (Nicht-ärztliche Praxisassistent/in)

VERAH = Care assistant in general practice (Versorgungsassistent/in in der Hausarztpraxis)

NRW-GPRN = North-Rhine Westphalia General Practice Research Network
